# Effects of helium and air inhalation on the innate and early adaptive immune system in healthy volunteers *ex vivo*

**DOI:** 10.1186/1479-5876-10-201

**Published:** 2012-09-24

**Authors:** Gezina TML Oei, Kirsten F Smit, Djai vd Vondervoort, Daniel Brevoord, Arjan Hoogendijk, Catharina W Wieland, Markus W Hollmann, Benedikt Preckel, Nina C Weber

**Affiliations:** 1 Laboratory of Experimental Intensive Care and Anesthesiology (L.E.I.C.A.) Academic Medical Centre, University of Amsterdam, Meibergdreef 9, Amsterdam, 1100 DD, The Netherlands; 2 Center for Infection and Immunity Amsterdam, Academic Medical Centre, University of Amsterdam, Meibergdreef 9, Amsterdam, 1100 DD, The Netherlands; 3 Center for Experimental and Molecular Medicine, Academic Medical Centre, University of Amsterdam, Meibergdreef 9, Amsterdam, 1100 DD, The Netherlands

**Keywords:** Noble gas, Side effects, Cell-mediated immunity, Ischemia-reperfusion injury, Whole blood stimulation

## Abstract

**Background:**

Helium inhalation protects myocardium, brain and endothelium against ischemia/reperfusion injury in animals and humans, when applied according to specific “conditioning” protocols. Before widespread use of this “conditioning” agent in clinical practice, negative side effects have to be ruled out. We investigated the effect of prolonged helium inhalation on the responsiveness of the human immune response in whole blood *ex vivo.*

**Methods:**

Male healthy volunteers inhaled 30 minutes heliox (79%He/21%O_2_) or air in a cross over design, with two weeks between measurements. Blood was withdrawn at T0 (baseline), T1 (25 min inhalation) and T2-T5 (1, 2, 6, 24 h after inhalation) and incubated with lipopolysaccharide (LPS), lipoteichoic acid (LTA), T-cell stimuli anti-CD3/ anti-CD28 (TCS) or RPMI (as control) for 2, 4 and 24 hours or not incubated (0 h). An additional group of six volunteers inhaled 60 minutes of heliox or air, followed by blood incubation with LPS and RPMI. Tumor necrosis factor-α (TNF-α), interleukin-1β (IL-1β), interleukin-6 (IL-6), interleukin-8 (IL-8), interferon-γ (IFN-γ) and interleukin-2 (IL-2) was analyzed by cytometric bead array. Statistical analysis was performed by the Wilcoxon test for matched samples.

**Results:**

Incubation with LPS, LTA or TCS significantly increased TNF-α, IL-1β, IL-6, IL-8, IFN-γ and IL-2 in comparison to incubation with RPMI alone. Thirty min of helium inhalation did not influence the amounts of TNF-α, IL-1β, IL-6, IL-8, IFN-γ and IL-2 in comparison to air. Sixty min of helium inhalation did not affect cytokine production after LPS stimulation.

**Conclusions:**

We conclude that 79% helium inhalation does not affect the responsiveness of the human immune system in healthy volunteers.

**Trial registration:**

Dutch Trial Register: http://www.trialregister.nl/ NTR2152

## Background

Helium, a colorless, odorless and tasteless gas with a lower density than air, was first used in patients with respiratory diseases [1]. Recently it was shown that beside volatile anesthetics (e.g. isoflurane, sevoflurane, desflurane) and the anesthetic noble gas xenon, the non-anesthetic noble gas helium also reduces ischemia-reperfusion injury when administered before (preconditioning) or after (postconditioning) organ ischemia [1,2]. Experimental studies in rabbits and rats suggested that helium conditioning protects myocardial and neuronal tissue against ischemia/reperfusion damage [3-8]. In a forearm model of ischemia-reperfusion injury in humans, helium preconditioning protected the endothelium against ischemic damage [9]. This suggests that helium can be a therapeutic agent against ischemia-reperfusion injury. However, before using helium as a “conditioning” agent in clinical settings, any negative effect on other organ systems such as the immune system should be ruled out.

Host immunity is classically divided into the innate and the adaptive immune system. The innate immune response involves monocytes, neutrophils, dendritic cells and macrophages but also parenchymal cells such as epithelial and endothelial cells. Upon danger sensing, secretion of cytokines and chemokines results in monocyte and neutrophil migration to inflamed tissues and antigen presentation. This initial and aspecific cascade induces secondary antigen specific events known as the adaptive immune system involving T- and B-lymphocytes. Volatile anesthetics and xenon exert immunomodulatory effects by affecting endothelial expression of adhesion molecules and the secretion of cytokines and chemokines [10] as well as lymphocytes [11-13].

We investigated whether helium breathing in healthy volunteers affects the ability of the immune system to respond to *ex vivo* stimulation of whole blood. For the innate arm, we measured the proinflammatory cytokines tumor necrosis factor-α (TNF-α), interleukin-1β (IL-1β) and interleukin-6 (IL-6) and chemokine interleukin-8 (IL-8) after stimulation with lipopolysaccharide (LPS) and lipoteichoic acid (LTA). To assess effects of helium on the adaptive immune system, interferon-γ (IFN-γ) and interleukin-2 (IL-2) production after T cell receptor specific stimulation was determined.

## Methods

The study was approved by the ethical committee of the Academic Medical Centre, Amsterdam ( http://www.trialregister.nl/ NTR2152) and was conducted in accordance with the International Conference on Harmonization on Good Clinical Practice Guidelines and the Declaration of Helsinki. After written informed consent, twelve healthy, non-smoking, male volunteers (age 22–35) were included and were asked to use no caffeine or alcohol containing drinks, and not to exert heavy physical exercise twelve hours before the experiment. Volunteers did not use any medication influencing the immune system, or were known to have any condition that could influence the immune system. A second group of 6 volunteers (age 19–31) inhaled 60 min of helium and air.

### Experimental design

Experiments were conducted in a crossover design in a quiet room with standardized circumstances. All participants underwent two experimental cycles: once with 30 or 60 minutes of heliox (79%He/21%O_2,_ BOC, Mordon, UK) inhalation using a non-invasive delivery system (Helontix^TM^vent, Linde Therapeutics, Eindhoven, The Netherlands) via a normal face mask with pressure support of 3 cm H_2_O, and once with air inhalation, with two weeks in between cycles. Venous blood was sampled at baseline (T0), at 25 min of inhalation (T1), or 1 (T2), 2 (T3), 6 (T4), or 24 h after inhalation (T5), respectively. C-reactive protein (CRP), leukocyte and lymfocyte counts were determined in ethylenediamine tetraaceticacid–anticoagulated blood, and heparin-anticoagulated blood was used for incubation with immune stimulants.

### Whole blood stimulation

After sampling, heparinized whole blood (0,5 ml) was diluted with an equal volume of RPMI-1640 (Invitrogen, Breda, the Netherlands) serving as control (CON), or RPMI-1640 containing LPS (Ultra pure LPS from Escheria coli 0111:B4, InvivoGen, San Diego, United States) in a final concentration of 200 ngml^-1^, RPMI-1640 containing LTA ( Purified LTA from Staphylococcus aureus, InvivoGen, San Diego, United States) in a final concentration of 20 μgml^-1^, or RPMI-1640 containing T-cell stimuli anti-CD3/anti-CD28 (TCS; murine monoclonal antibodies CLB-T3/3 against the CD3 molecular complex and CLB-CD28/1 against the T-cell differentiation antigen CD28, provided by dr. R. van Lier, Academic Medical Centre, Amsterdam, The Netherlands) in a final concentration of 10 μgml^-1^, respectively. Incubation was done in aliquots of 0,5 ml in sterile tubes (Sarstedt, Etten-Leur, the Netherlands) at 37°C for 0, 2, 4 or 24 hours, all performed in duplicate. After incubation, plasma was prepared by centrifugation at 1200 RPM for 10 minutes at 4°C. Plasma was stored at −20°C until further analysis.

Established in our research institution by the laboratory of van der Poll, the use of whole-blood cultures now is a widely used method to screen for influences of treatments on the immune response [14]. The whole blood induced cytokine production by specific bacterial antigens has important advantages. In this system, cell populations that are important for the defense against pathogenic organisms (e.g. neutrophils, monocytes, and lymphocytes) and soluble factors like complement, antibodies, and other serum components can interact thus resembling the *in vivo* situation. LPS is a major constituent of the cell wall of Gram-negative bacteria, LTA a constituent of the cell wall of Gram-positive bacteria. Using both stimuli thereby covers a broad range of microbial agents and the resulting activation of monocytes and neutrophils induces synthesis of proinflammatory cytokines such as TNF-α, IL-1β and IL-6 and chemokine IL-8 [15-17]. These cytokines and chemokines are in turn able to activate T lymphocytes.

In contrast to pro-inflammatory cytokines that will peak within a few hours after exposure to antigens, the T-cell mediated response usually peaks later and can be monitored by the production of the typical cytokines that reflect T cell function, among which IFN-γ and IL-2. We studied T-cell function by specific activation of the T cell receptor through application of a combination of antibodies directed against CD3 and CD28 [18,19].

### Cytokine and chemokine measurement by multiplex bead-based immunoassays

Plasma TNF-α, IL-1β, IL-6, IL-8, IFN-γ and IL-2 concentrations were measured simultaneously by cytometric bead array (CBA), a flow cytometry based fluorescence detection of antibody-coated beads (‘Human Inflammatory Cytokine Kit’ and ‘Human Th1/Th2 cytokine kit’, BD Biosciences, Breda, the Netherlands). For measurement and analysis of cytokines, we used the fluorescent activated cell sorter FACSCalibur (BD Biosciences, Breda, The Netherlands) with BD FACSComp and BD CellQuest software.

### Statistical analysis

**Figure 1:**
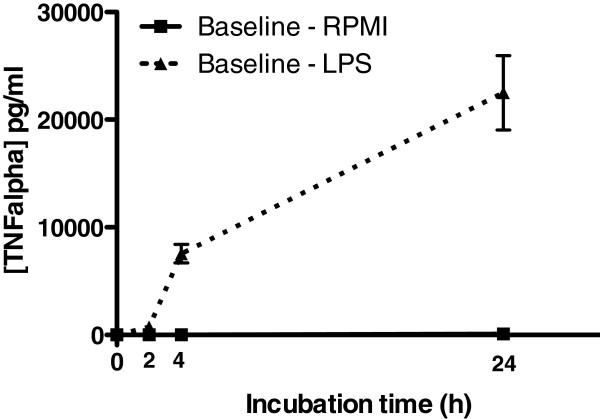
**TNF-α levels in plasma after 0,2,4, and 24 hours of incubation with LPS in RPMI (dashed line) or RPMI alone.** Blood was sampled at time point T0 (baseline), before 30 min of helium or air inhalation. Data (n = 24 per group) shown are means +/− SEM.

**Figure 2:**
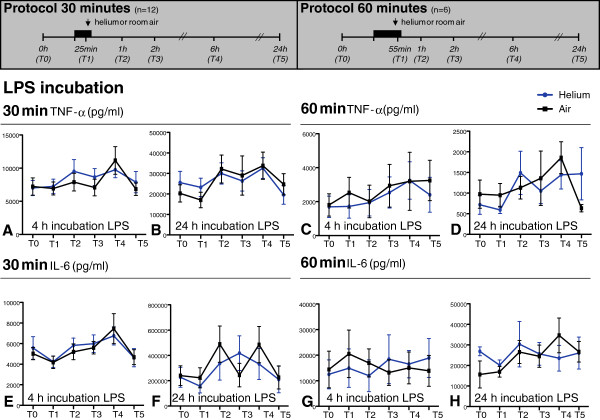
**TNF-α (panel A-D) and IL-6 (panel E-H) levels in plasma after 4 and 24 hours incubation with LPS.** Time points on y-axis represent blood sampling time points shown in the experimental protocols above. Panels **A**, **B**, **E** and **F** show 30 minutes of inhalation of helium and air, panels **C**, **D**, **G** and **H** 60 minutes. Data shown are means +/− SEM. Experimental protocol is shown above; T0: baseline, T1: at 25 min inhalation, T2: 1 h after inhalation, T3: 2 h after inhalation, T4: 6 h after inhalation, T5: 24 h after inhalation.

**Figure 3:**
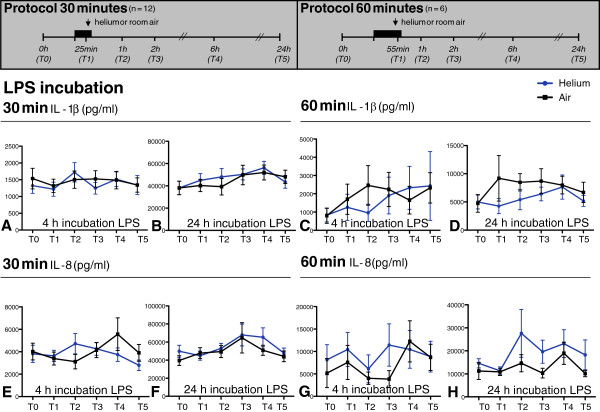
**IL-1β (panels A-D) and IL-8 (panels E-H) levels in plasma after 4 and 24 hours incubation with LPS.** Time points on y-axis represent blood sampling time points shown in the experimental protocols above. Panels **A**, **B**, **E** and **F** show 30 minutes of inhalation of helium and air, panels **C**, **D**, **G** and **H** 60 minutes. Data shown are means +/− SEM. Experimental protocol is shown above; T0: baseline, T1: at 25 min inhalation, T2: 1 h after inhalation, T3: 2 h after inhalation, T4: 6 h after inhalation, T5: 24 h after inhalation.

**Figure 4:**
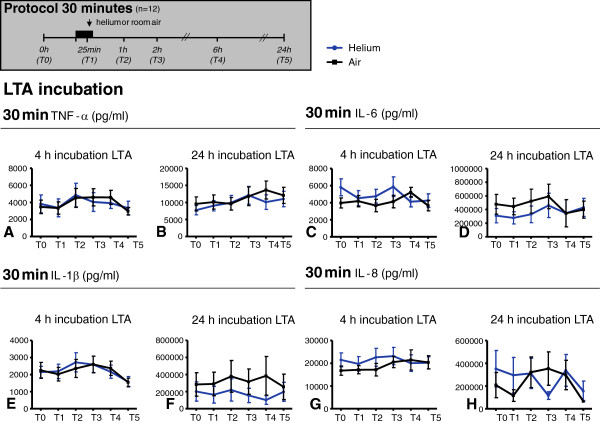
**TNF-α (panel A-B), IL-6 (panel C-D), IL-1β (panel E-F) and IL-8 (panel G-H) levels in plasma after 4 and 24 hours incubation with LTA.** Time points on y-axis represent blood sampling time points shown in the experimental protocols above. Panels show 30 minutes of inhalation. Data shown are means +/− SEM. Experimental protocol is shown above; T0: baseline, T1: at 25 min inhalation, T2: 1 h after inhalation, T3: 2 h after inhalation, T4: 6 h after inhalation, T5: 24 h after inhalation.

Normal distribution of the data was tested with the Kolmogorov-Smirnov test. As data were not normally distributed, differences between helium and air groups were tested by the Wilcoxon test for paired measurements and considered significant if p < 0.05. All data in Figures 1, 2, 3 and 4 are shown as mean ± SEM. An overview of all p-values (p), number of helium-air pairs included in the Wilcoxon test at each time point (n)*, number of experiments in the helium group (n He), and the air group (n Air) are given in Table 1. The table contains values for all figures included in the manuscript (Figure 1, 2, 3 and 4). N-numbers vary in some cases due to technical problems with cytokine and chemokine measurements.

**Table 1:** P- and n- values


**Figure 2**	A (TNF-α, 30 min He, 4 h LPS)				B (TNF-α, 30 min He, 24 h LPS)				C (TNF-α, 60 min He, 4 h LPS)				D (TNF-α, 60 min He, 24 h LPS)			
	p	n	n He	n Air	p	n	n He	n Air	p	n	n He	n Air	p	n	n He	n Air
T0	0.68	12	12	12	0.42	12	12	12	0.84	6	6	6	0.53	6	6	6
T1	0.91	12	12	12	0.73	12	12	12	0.44	6	6	6	0.35	6	6	6
T2	0.73	12	12	12	0.52	12	12	12	0.06	5	6	5	0.50	6	6	6
T3	0.38	12	12	12	0.79	12	12	12	0.81	5	5	6	0.72	6	6	6
T4	0.27	12	12	12	0.73	12	12	12	0.44	5	5	6	0.53	6	6	6
T5	0.52	12	12	12	0.68	12	12	12	0.56	6	6	6	0.70	6	6	6
	**E** (IL-6, 30 min He, 4 h LPS)				**F** (IL-6, 30 min He, 24 h LPS)				**G** (IL-6, 60 min He, 4 h LPS)				**H** (IL-6, 60 min He, 24 h LPS)			
	p	n	n He	n Air	p	n	n He	n Air	p	n	n He	n Air	p	n	n He	n Air
T0	0.91	12	12	12	0.65	10	10	11	1.00	6	6	6	0.09	6	6	6
T1	0.83	11	11	12	0.08	11	11	12	0.16	6	6	6	0.44	6	6	6
T2	0.42	12	12	12	0.62	10	10	12	0.44	6	6	6	0.84	6	6	6
T3	0.91	12	12	12	0.11	11	11	11	1.00	4	4	5	1.00	6	6	6
T4	0.76	11	12	11	0.23	10	10	12	1.00	5	6	5	0.44	6	6	6
T5	1.00	12	12	12	0.77	11	11	11	0.69	5	6	5	0.69	6	6	6
**Figure 3**	**A** (IL-1β, 30 min He, 4 h LPS)				**B** (IL-1β, 30 min He, 24 h LPS)				**C** (IL-1β, 60 min He, 4 h LPS)				**D** (IL-1β, 60 min He, 24 h LPS)			
	p	n	n He	n Air	p	n	n He	n Air	p	n	n He	n Air	p	n	n He	n Air
T0	0.30	12	12	12	0.28	12	12	12	1.00	6	6	6	1.00	5	6	5
T1	0.42	12	12	12	0.42	12	12	12	0.56	6	6	6	0.44	6	6	6
T2	0.34	12	12	12	0.30	12	12	12	0.19	6	6	6	0.31	6	6	6
T3	0.13	12	12	12	0.97	12	12	12	0.44	5	5	5	0.44	6	6	6
T4	0.70	11	12	11	0.97	12	12	12	1.00	6	6	6	1.00	6	6	6
T5	1.00	12	12	12	0.34	12	12	12	0.69	6	6	6	0.69	6	6	6
	**E** (IL-8, 30 min He, 4 h LPS)				**F** (IL-8, 30 min He, 24 h LPS)				**G** (IL-8, 60 min He, 4 h LPS)				**H** (IL-8, 60 min He, 24 h LPS)			
	p	n	n He	n Air	p	n	n He	n Air	p	n	n He	n Air	p	n	n He	n Air
T0	0.34	12	12	12	0.46	10	10	10	0.22	6	6	6	0.22	6	6	6
T1	0.34	12	12	12	0.47	9	10	9	0.31	6	6	6	0.84	6	6	6
T2	0.20	12	12	12	0.31	10	10	10	0.56	6	6	6	0.44	6	6	6
T3	0.51	12	12	12	0.43	11	11	11	0.44	6	6	6	0.16	6	6	6
T4	0.91	11	12	11	0.50	10	11	10	0.84	6	6	6	0.44	6	6	6
T5	0.97	12	12	12	0.13	10	10	11	1.00	6	6	6	0.84	6	6	6
**Figure 4**	**A** (TNF-α, 30 min He, 4 h LTA)				**B** (TNF-α, 30 min He, 24 h LTA)				**C** (IL-6, 30 min He, 4 h LTA)				**D** (IL-6, 30 min He, 24 h LTA)			
	p	n	n He	n Air	p	n	n He	n Air	p	n	n He	n Air	p	n	n He	n Air
T0	0.90	12	12	12	0.68	12	12	12	0.24	11	12	11	0.55	11	11	12
T1	0.68	12	12	12	0.76	12	12	12	0.89	11	12	11	0.02*	11	11	12
T2	0.73	12	12	12	0.79	12	12	12	0.05	11	11	11	0.64	11	11	12
T3	0.30	12	12	12	0.94	12	12	12	0.24	11	12	11	0.24	11	11	12
T4	0.20	11	12	11	0.38	11	12	11	0.07	11	11	12	0.05	11	11	12
T5	0.97	12	12	12	0.91	12	12	12	0.41	11	12	12	0.32	11	11	12
	**E** (IL-1β, 30 min He, 4 h LTA)				**F** (IL-1β, 30 min He, 24 h LTA)				**G** (IL-8, 30 min He, 4 h LTA)				**H** (IL-8, 30 min He, 24 h LTA)			
	p	n	n He	n Air	p	n	n He	n Air	p	n	n He	n Air	p	n	n He	n Air
T0	0.89	11	11	12	1.00	11	11	12	0.34	12	12	12	0.19	8	8	10
T1	0.62	12	12	12	0.44	11	11	12	0.34	12	12	12	0.63	8	9	8
T2	0.34	12	12	12	0.31	11	11	12	0.20	12	12	12	0.74	10	10	11
T3	0.97	12	12	12	0.44	11	11	12	0.51	12	12	12	0.81	9	9	10
T4	0.38	12	12	12	1.00	11	11	12	0.91	11	12	11	0.07	11	11	11
T5	1.00	11	11	11	0.69	11	11	12	0.97	12	12	12	0.20	11	11	11
**Figure 5**	**A** (IL-2, 30 min He, 4 h TCS)				**B** (IL-2, 30 min He, 24 h TCS)				**C** (IFN**-**Υ, 30 min He, 4 h TCS)				**D** (IFN**-**Υ, 30 min He, 24 h TCS)			
	p	n	n He	n Air	p	n	n He	n Air	p	n	n He	n Air	p	n	n He	n Air
T0	0.76	11	11	11	0.83	11	11	11	0.92	11	11	11	0.16	11	11	11
T1	0.83	11	11	11	0.97	11	11	11	1.00	11	11	11	0.16	10	11	10
T2	0.58	11	11	11	0.46	11	11	11	0.01*	11	11	11	0.91	10	10	10
T3	0.76	11	11	11	0.85	11	11	11	0.38	11	11	11	0.16	10	12	10
T4	0.28	11	11	11	0.10	11	11	11	0.13	11	11	11	0.85	10	12	10
T5	0.28	11	11	11	0.58	11	11	11	1.00	11	11	11	0.01*	10	11	10

## Results

### Helium inhalation does not influence leukocyte and lymphocyte counts

At baseline, there was no difference in C-reactive protein between the 30 min inhalation groups; 1.6 ± 0.4 (mg/l) and 2.3 ± 0.6 (mg/l) in the helium and air group respectively (p > 0.05). In the 60 min inhalation group, there was no difference at baseline in C-reactive protein either: 0.92 mg/l in the helium group versus 0.82 mg/l in the air group. Table 2 shows leukocyte and lymphocyte counts; no differences could be detected between heliox and air inhalation at baseline, 2 or 24 hours after inhalation (p > 0.05), for both the 30 min and the 60 min inhalation group. The data are shown as mean ± SEM, no statistic differences between groups (p > 0.05).

**Table 2:** Leukocyte and lymphocyte counts

**30 min inhalation**	**Leukocytes (10**^**9**^**/l) (n = 11–12)**		**Lymfocytes (10**^**9**^**/l) (n = 10–12)**	
**Time point**	**Heliox**	**Room air**	**Heliox**	**Room air**
T0	5,76 ± 0,38	5,54 ± 0,38	1.96 ± 0.18	1.97 ± 0.08
T3	5,79 ± 0,34	5,78 ± 0,44	1.77 ± 0.13	1.88 ± 0.12
T5	5,79 ± 0,41	5,53 ± 0,34	2.07 ± 0.08	1.93 ± 0.10
**60 min inhalation**	**Leukocytes (10**^**9**^**/l) (n =6)**		**Lymfocytes (10**^**9**^**/l) (n = 4–6)**	
**Time point**	**Heliox**	**Room air**	**Heliox**	**Room air**
T0	5,55 ± 0,29	5,83 ± 0,21	1.88 ± 0.24	1.96 ± 0.24
T3	6,28 ± 0,55	7,02 ± 0,60	1.99 ± 0.21	2.11 ± 0.26
T5	5,57 ± 0,36	5,65 ± 0,48	1.77 ± 0.27	1.73 ± 0.19

### *Ex vivo* stimulation of whole blood with LPS, LTA or TCS significantly increased TNF-α, IL-1β, IL-6, IL-8, IFN-γ and IL-2 in comparison to incubation with RPMI alone

After 2, 4 and 24 hours of incubation with LPS, the amount of TNF-α was significantly higher in comparison to incubation with RPMI alone, also see Figure 1.

After 2, 4 and 24 hours of incubation with LPS or LTA, the amount of TNF-α, IL-1β, IL-6 and IL-8 (pg/ml) was significantly higher in comparison to incubation with RPMI alone (data not shown). After 2, 4 and 24 hours of incubation with TCS, the amount of IFN-γ, and IL-2 was significantly higher in comparison to incubation with RPMI alone (data not shown), indicating that the used immune agents were able to induce an adequate immune response.

### Helium inhalation for 30 and 60 minutes does not influence inflammatory cytokine and chemokine levels in whole blood after *ex vivo* incubation with LPS, in comparison to inhalation of air

Thirty or 60 min of helium inhalation did not affect the amount of TNF-α, IL-6, IL-1β and IL-8 (pg/ml) after 0, 2 (data not shown), 4 and 24 hours of incubation of whole blood with LPS in comparison to room air at all time points (p > 0.05). Figures 2 (TNF-α, IL-6) and 3 (IL-1β and IL-8) show cytokine levels after 4- and 24-hour stimulations with LPS.

### Helium inhalation for 30 min does not influence inflammatory cytokine and chemokine levels in whole blood after *ex vivo* incubation with LTA, in comparison to inhalation of air

After 0, 2, 4 and 24 hours of incubation with LTA the amount of TNF-α, IL-1β, and IL-8 (pg/ml) did not differ between heliox and air groups (p > 0.05). The amount of IL-6 after 24 hours of incubation with LTA was similar after heliox or air inhalation at baseline (T0), 1 (T2), 2 (T3), 6 (T4) or 24 (T5) hours after inhalation (p > 0.05), but differed significantly after 25 minutes of helium inhalation compared to air (T1). Figure 4 shows cytokine levels after 4 and 24 incubation with LTA, data at 0 and 2 hours are not shown.

### Helium inhalation for 30 min does not influence levels of inflammatory cytokines IL-2 and IFN-γ excreted in whole blood after *ex vivo* incubation with T-cell stimuli anti-CD3/anti-CD28, in comparison to inhalation of room air

**Figure 5:**
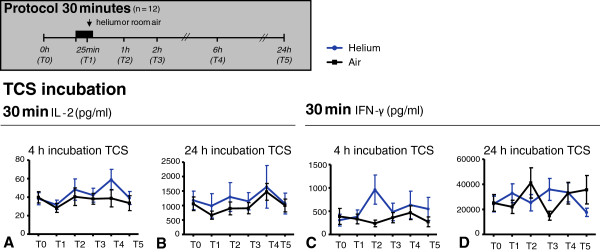
**IL-2 (panel A-B) and IFN-γ (panel C-D) levels in plasma after 4 and 24 hours incubation with TCS anti-CD3/28.** Time points on y-axis represent blood sampling time points shown in the experimental protocols above. Panels show 30 minutes of inhalation. Data shown are means +/− SEM. Experimental protocol is shown above; T0: baseline, T1: at 25 min inhalation, T2: 1 h after inhalation, T3: 2 h after inhalation, T4: 6 h after inhalation, T5: 24 h after inhalation.

After 0, 2, 4 and 24 hours of incubation with anti-CD3/antiCD28, the amount of IFN-γ and IL-2 (pg/ml) was not affected by helium inhalation in comparison to room air at T0, T1, T2, T4, or T5 (p > 0.05), except for statistically different IFN-γ levels at two time points. After 4 hours of incubation, the amount of IFN-γ 1 hour after 30 min of helium inhalation (T2) was statistically higher in comparison to air inhalation. After 24 hours of incubation, the amount of IFN-γ 24 hours after air inhalation was significantly different in comparison to helium inhalation. IFN-γ and IL-2 levels after 30 min of helium/air inhalation are shown in Figure 5.

## Discussion

Our study suggests that prolonged inhalation of helium does not affect the ability of the innate and early adaptive immune system to respond to immune stimuli LPS, LTA or anti-CD3/anti-CD28 *ex vivo.* TNF-α, IL-1β, IL-6, IL-8, IFN-γ and IL-2 levels did not differ at various time points before and after helium inhalation compared to air inhalation. These results are of interest for a broad field, as the use of helium-oxygen mixtures for respiratory disease or with the purpose of cell protection against ischaemia/reperfusion injury in the critical care unit and the operating theatre might expand.

Two *in vivo* studies using a forearm model of ischaemia-reperfusion injury investigated the protective effect of helium inhalation on endothelial function and additionally looked at systemic immune parameters [9,20]. Our results are in line with results found in the first study, in which similar concentrations of helium/oxygen were used [9]. This study showed that application of three 5-minute cycles of helium (79%He/21%O_2_) interspersed with 5 min of air breathing before the ischemic episode induces preconditioning in human endothelium [9]. To investigate the influence on the innate immune system, venous blood was collected at the non-injured arm for analysis of systemic levels of adhesion molecules sVCAM-1, sICAM-1, E-selectin, and proinflammatory cytokines IL-1β and IL-8 at 10 minutes and 3 hours after reperfusion. No effects of forearm ischaemia/reperfusion or helium conditioning could be found on all of these parameters, suggesting no net effect of helium on the investigated immune parameters [9].

In contrast, Lucchinetti and colleagues found immunomodulatory effects after 35 minutes of helium breathing in a concentration of 50%He/50%O_2_[20]. Venous blood was taken from the injured arm at baseline, and after 5, 10 and 30 minutes of reperfusion to investigate proinflammatory markers on leukocytes. A decrease of CD11b on monocytes at 10 and 30 minutes of reperfusion and a decrease of ICAM-1 on monocytes at 5 minutes of reperfusion was found under helium inhalation in comparison to control. Although no effects on other markers were found, these findings implicated a net negative effect of helium inhalation on systemic immune parameters [20]. However, differences in duration and concentration of the inhaled helium/oxygen mixtures make a direct comparison between the aforementioned studies difficult. Another difference is the proximity of the induced tissue damage (forearm ischemia-reperfusion) and the point of blood sampling for analysis of immune parameters. In the latter study, blood collection took place from the injured arm – closer to the site of injury - in contrast to the first study in which blood collection took place from the non-injured arm [9,20]. It might be possible that the immunomodulatory effect of helium can only be found locally or that helium exerts anti-inflammatory effects only when tissue damage is present. An example is the anti-inflammatory effect of helium in comparison to nitrox breathing that was found in a pig model of acute lung injury [21]. Anti-inflammatory effects were shown in lung tissue as a reduction of pro-inflammatory cytokine IL-8 and myeloperoxidase, a measure for neutrophil activity. The big difference with the current study is the lack of tissue damage at the time of helium breathing: stimulation of the immune system is done after helium breathing and blood sampling. This provides an objective way of assessing the immunomodulatory characteristics of this noble gas *per se.*

The main rationale behind the present study was to investigate whether the use of helium gas against organ ischemia/reperfusion injury may have detrimental effects on the immune response. Ischemic and pharmacologic conditioning protocols described in the literature normally do not exceed a total of 30 minutes of intervention [22-24]. Therefore, 30 minutes of helium inhalation resembles a clinically relevant time frame of gas application. Investigation of the innate immune response after 60 min of helium and air inhalation was done in an additional group, to rule out that prolonged inhalation did exert effects. Furthermore, a concentration of 79% helium is the maximum from a clinical point of view, although a variety of lower helium concentrations have also been used [25]. Higher percentages of helium lead inevitably to hypoxic gas mixtures. It is highly unlikely that lower concentrations have detrimental effects on the immune system, when a higher dose does not.

In this study, we mainly focused on pro-inflammatory components of the immune system. However, in some cases it is not so clear whether cytokines exhibit purely pro- or anti-inflammatory actions, such as IL-2. This cytokine has pro-inflammatory effects, but might play an anti-inflammatory role in diabetes [26]. The finding that no effects on pro-inflammatory components were found suggests that no net-effect of helium inhalation on the immune response exists. It has to be noted that clinical outcome of infections is the result of a balance in quantity and time course of pro- and anti-inflammatory components. Therefore, several other anti-inflammatory components of the immune system, as well as other constituents of the innate and early adaptive immune system still need further investigation. In our study we did not investigate cytokine production in fractionated blood leukocytes. Instead, we investigated total amounts of cytokines in whole blood, as this model represents a condition in which many of the physiologically present cellular interactions remain intact. Given the fact that leukocyte counts did not differ between groups at the different time points either, we consider the lack of difference in cytokine levels between groups a good reflection of the unaltered immune status after helium inhalation.

Whole blood stimulation is a widely known model used for various goals. In a recent study it was shown that *ex vivo* stimulation of whole blood with pathogenic Leptospira induced a cytokine response [27]. The whole blood stimulation model was also used in another study to show that erythromycin infusion in healthy volunteers reduces IL-8 production after *ex vivo* stimulation with Streptococcus pneumonia [28]. Stress-related suppression of cytokine production after whole blood incubation with LPS was shown in a study in which 20 male, healthy volunteers were exposed to bungee jumping [29]. In this study it was shown that bungee jumping was associated with higher epinephrine, norepinephrine and cortisol levels, but also with increased leukocytes. Nevertheless, the amount of TNF-α and IL-8 levels after *ex vivo* stimulation with LPS was decreased [29].

Despite the evidence for the applicability of our used model, a limitation of the study is the difficulty of proving absence of an effect while 3 significant differences between helium and air inhalation were found. However, with a total of 168 tests being performed in total, 8 can be significant by chance alone. Furthermore, significant findings at solely one time point of one cytokine after one stimulation type are not likely to reflect a clinically relevant effect of the intervention. As can be seen in the figures, lines of helium and air inhalation intersect at random time points, suggesting that even when an effect seems to be there it does not persist over time.

A second limitation of the study concerns the investigation of healthy, male volunteers only. The target population for helium-induced organ protection often suffers from comorbidity, which might be of influence on the innate immune response to *ex vivo* stimulation. We have chosen to use a model in which possible confounders by comorbidities were excluded. From literature it is known that sex differences exist in immune defense capacity and cytokine production [30]. To minimize the influence of this possible confounder we have chosen to investigate males only.

## Conclusions

The results of the present study indicate that inhalation of helium for 30 and 60 minutes does not affect leukocyte counts and does not have detrimental effects on the ability to evoke an adequate immune response in healthy volunteers after *ex vivo* whole blood stimulation with LPS, LTA and anti-CD3/CD28. These findings have implications for the use of helium as a conditioning agent in clinical practice, as it seems unlikely that helium affects innate immunity.

## Abbreviations

LPS: Lipopolysaccharide; LTA: Lipoteichoic acid; TCS: T-cell stimuli (anti-CD3/ anti-CD28); TNF-α: Tumor necrosis factor-alpha; IL-1β: Interleukin-1beta; IL-6: Interleukin-6; IL-8: Interleukin-8; IFN-γ: Interferon-gamma and IL-2 interleukin-2.

## Competing interests

The authors declare that they have no competing interests.

## Authors’ contributions

GO was involved in the conception and design of the study; collected, analyzed and interpreted the data and wrote the manuscript. KS contributed to conception and design of the study and collection of the data. DV contributed to collection of the data. DB contributed to collection of the data. AH contributed to analysis of the data. CW was involved in the conception and design of the study and supervised writing of the manuscript. MH was involved in the conception and design of the study and supervised writing of the manuscript. BP was involved in the conception and design of the study and supervised writing of the manuscript. NW was involved in the conception and design of the study and supervised writing of the manuscript. All authors read and approved the final manuscript.

## Funding

Support was provided from institutional and/or departmental sources and from an award from the Dutch Society of Anesthesiologists, NVA Utrecht, The Netherlands to G.T.M.L. Oei. C.W. Wieland was supported by a grant from The Netherlands Organization for Scientific Research.
